# Osteoimmunology of Bone Loss in Inflammatory Rheumatic Diseases

**DOI:** 10.3389/fimmu.2019.00679

**Published:** 2019-04-03

**Authors:** Fabienne Coury, Olivier Peyruchaud, Irma Machuca-Gayet

**Affiliations:** ^1^INSERM, UMR1033 LYOS, Lyon, France; ^2^University Claude Bernard Lyon I, Lyon, France; ^3^Department of Rheumatology, Lyon Sud Hospital, Lyon, France

**Keywords:** inflammatory rheumatic diseases, rheumatoid arthritis, spondyloarthritis, bone erosion, inflammatory bone loss, osteoclast

## Abstract

Over the past two decades, the field of osteoimmunology has emerged in response to a range of evidence demonstrating the reciprocal relationship between the immune system and bone. In particular, localized bone loss, in the form of joint erosions and periarticular osteopenia, as well as systemic osteoporosis, caused by inflammatory rheumatic diseases including rheumatoid arthritis, the prototype of inflammatory arthritis has highlighted the importance of this interplay. Osteoclast-mediated resorption at the interface between synovium and bone is responsible for the joint erosion seen in patients suffering from inflammatory arthritis. Clinical studies have helped to validate the impact of several pathways on osteoclast formation and activity. Essentially, the expression of pro-inflammatory cytokines as well as Receptor Activator of Nuclear factor κB Ligand (RANKL) is, both directly and indirectly, increased by T cells, stimulating osteoclastogenesis and resorption through a crucial regulator of immunity, the Nuclear factor of activated T-cells, cytoplasmic 1 (NFATc1). Furthermore, in rheumatoid arthritis, autoantibodies, which are accurate predictors both of the disease and associated structural damage, have been shown to stimulate the differentiation of osteoclasts, resulting in localized bone resorption. It is now also evident that osteoblast-mediated bone formation is impaired by inflammation both in joints and the skeleton in rheumatoid arthritis. This review summarizes the substantial progress that has been made in understanding the pathophysiology of bone loss in inflammatory rheumatic disease and highlights therapeutic targets potentially important for the cure or at least an alleviation of this destructive process.

## Introduction

The close relationship between the immune and bone systems has long been noted since pioneering work on soluble immune cell-derived osteoclast-activating factors performed in the early 1970s (1, 2) and was termed osteoimmunology (3). The most significant osteoimmunological example arose from the observation of osteoclast-mediated bone loss in inflammatory rheumatic diseases. Inflammatory rheumatic diseases encompass more than 100 heterogeneous multisystem disorders which can affect joints and lead to disability. However, rheumatoid arthritis (RA) and the spondyloarthritis group (SpA) are the most common inflammatory rheumatic diseases that preferentially affect joints and cause tenderness, swelling, and destruction of joints. Consequently, in this review, we will confine the term “inflammatory rheumatic diseases” to these particular diseases. SpA, also termed “seronegative” as they do not produce rheumatoid factor nor the anti-citrullinated peptide antibodies (ACPA) observed in RA, represent a group of diseases with common genetic and clinical features, including ankylosing spondylitis (AS), reactive arthritis, psoriatic arthritis (PsA), and SpA associated with inflammatory bowel disease.

RA is considered to be the prototype of destructive inflammatory arthritis with bone loss at sites of articular and peri-articular inflammation. SpA also causes inflammation of the axial skeleton and extra-articular entheses leading to not only bone degradation but also to ectopic bone formation—which in some cases can even lead to bony ankylosis of the joint. Genetic and experimental evidence has associated the activation of IL23-IL17 axis with inflammation and entheseal new bone. The ectopic bone formation aspect of SpA will not be discussed further, as herein review focus is restricted to bone loss, formation is reviewed elsewhere (4). This dissimilarity in the anatomical sites of bone affected and in bone formation patterns highlights the differences in pathophysiological mechanisms involved in these conditions.

Herein, we briefly highlight the key concepts and recent advances in the osteoimmunology field within the context of bone loss in inflammatory rheumatic diseases.

## Differential Bone Loss in Inflammatory Rheumatic Diseases

Three forms of bone loss have been identified in patients with inflammatory rheumatic diseases: localized bone loss with erosion, periarticular osteopenia, and generalized bone loss (Table 1).

**Table 1:** Common features and differences in bone loss between SpA and RA.

	**SpA**	**RA**
	**(AS, PsA, reactive arthritis)**	
Erosions	•DIP, PIP joints •Evenly distributed •Small, Ω or tubule-shaped •Poorly demarcated •Periarticular site •Association with enthesitis and bone formation	•MTP, MCP, PIP, and wrist joints •Radial sites •U-shaped •Neatly demarcated •Joint margins •No association with enthesitis and bone formation
Periarticular osteopenia	•absent	•May precede bone erosion
Generalized bone loss	•Axial skeleton •Vertebral fractures •Association with ectopic new bone formation	•Axial and appendicular skeleton •Vertebral and non-vertebral fractures •No association with ectopic new bone formation
Bone remodeling	•↑ Bone resorption	•↑ Bone resorption •↓ bone formation

*DIP, distal interphalangeal; MTP, metatarsophalangeal; MCP, metacarpophalangeal; PIP, proximal interphalangeal. RA erosions, Neatly demarcated and located at joint margins where the inflamed synovium is in direct contact with bone, erosions in RA are U-shaped and observed predominantly in metacarpophalangeal / metatarsophalangeal and proximal interphalangeal joints with a strong preponderance for radial sites; PsA erosion, Poorly demarcated, smaller in size and depth, Ω or tubule-shaped, and are more evenly distributed. They are located in the periarticular site in proximal and distal interphalangeal joints and are closely associated with bone formation*.

Although cortical bone erosion revealed by radiography is commonly considered to be a hallmark of RA, it can also be observed in SpA as well as other rheumatic diseases such as gout or osteoarthritis—with a distinct radiographic appearance and location. Erosion begins early in inflammatory rheumatic diseases, even prior to the clinical onset of arthritis: erosion has been described in ACPA-positive healthy subjects (5). For long considered as being less destructive than RA, PsA is much more aggressive than previously thought. Essentially, about 20% of PsA patients develop a mutilating form of arthritis and 40–60% of PsA patients develop erosions in the first 2 years of the disease (6). Usually considered to be irreversible, bone erosion is a key outcome in inflammatory rheumatic diseases and correlates with disease severity and functional deterioration. The radiographic assessment of bone erosion is the ≪ gold standard ≫ for diagnosis, in daily clinical practice as well as in randomized controlled clinical trials of disease-modifying antirheumatic drugs, but is challenging. The development of more sensitive and reproducible analysis using ultrasound, magnetic resonance imaging or high-resolution peripheral quantitative computer tomography would be a promising development for erosion detection and monitoring in daily clinical practice. Periarticular trabecular bone is also altered in RA likely with similar mechanisms involved in generalized bone loss. Radiographic periarticular osteopenia is one of the earliest radiological manifestations and may precede bone erosion or joint space narrowing in RA (7). In contrast, it appears that there is no periarticular bone loss in early PsA (8).

Secondary systemic osteopenia or osteoporosis involving the axial and appendicular skeleton remote from synovial inflammation is an important co-morbidity in inflammatory rheumatic diseases. In effect, the prevalence of densitometric osteoporosis in RA patients is increased about two fold compared with the general population and is responsible for a risk of both vertebral and non-vertebral fractures (9). Although patients with SpA have radiographic evidence of ectopic new bone formation, many present evidences of marked osteopenia, and osteoporosis in the spine that is associated with a high prevalence of vertebral fractures—even in early axial SpA (10, 11). Inflammation is the major mechanism involved in bone loss in inflammatory rheumatic diseases. Proinflammatory cytokines increase osteoclast activation and subsequent bone resorption in both rheumatic disease types (12) but inhibit bone formation only in RA (13, 14). As a consequence, treatment with TNF-blockers both in RA and SpA has been shown to improve skeletal remodeling (15, 16). Apart from inflammation, others factors play a role such as the adverse skeletal effects of corticosteroids used to treat these diseases and immobility, due to painful joints, muscle weakness, and spine ankylosis—although bone loss is observed well-before the development of spinal immobility (17–19).

## Osteoclast Differentiation and Function in Inflammatory Rheumatic Diseases

Osteoclasts are responsible for bone erosion and have been identified at sites of focal erosion at the pannus-bone interface both in RA patients (20, 21) and animal models of arthritis (22–26). This role was definitively demonstrated by osteoclast-deficient mouse models of arthritis which were shown to be fully protected from bone erosion (25, 26). Osteoclasts are multinucleated bone resorbing cells which originate from the fusion of mononucleated cells belonging to the myeloid lineage in the presence of macrophage colony-stimulating factor (M-CSF) and Receptor Activator of Nuclear factor-κB Ligand (RANKL). Osteoclast formation is governed by a regulatory triad, the receptor activator of NF-κB (RANK), its ligand RANKL and a decoy receptor osteoprotegerin (OPG) also known as osteoclastogenesis inhibitory factor. OPG binds to RANKL hampering RANK-RANKL interaction, though RANKL/OPG ratio determines osteoclast number, lifespan and activity. Activation of RANK on mononuclear osteoclast precursors initiates a transcriptional cascade culminating in osteoclast differentiation. Interestingly, transcription factors important for osteoclast differentiation are key regulators of immune responses—such as NF-κB and nuclear factor of activated T cells cytoplasmic 1 (NFATc1). RANKL signaling in osteoclasts is strengthened by the synergistic activation of Immunoreceptor tyrosine-based activation motif (ITAM)-containing proteins, DNAX-activating protein of 12 kDa (DAP12) and Fc gamma receptor (FcRγ) (27, 28).

RANKL expression is high in synovial tissue from RA, PsA, and SpA peripheral joint disease patients (29–32). Treatment with non-biologic disease-modifying anti-rheumatic drugs (DMARDs) or glucocorticoids decreases the RANKL/OPG ratio in RA synovium and is satisfyingly associated with improved radiographic scores (17, 33). In addition, pharmacological inhibition of osteoclasts either by bisphosphonate zolendronic acid, or denosumab, a RANKL-specific monoclonal blocking antibody, also demonstrated some efficacy in impairing the progression of bone erosion in both arthritic mice and RA patients (34–38). However, these anti-resorptive drugs targeting osteoclasts are inadequate because they also alter physiological bone remodeling, necessitating the discovery of new targets.

## Role of T cells in osteoclastogenesis

**Figure 1:**
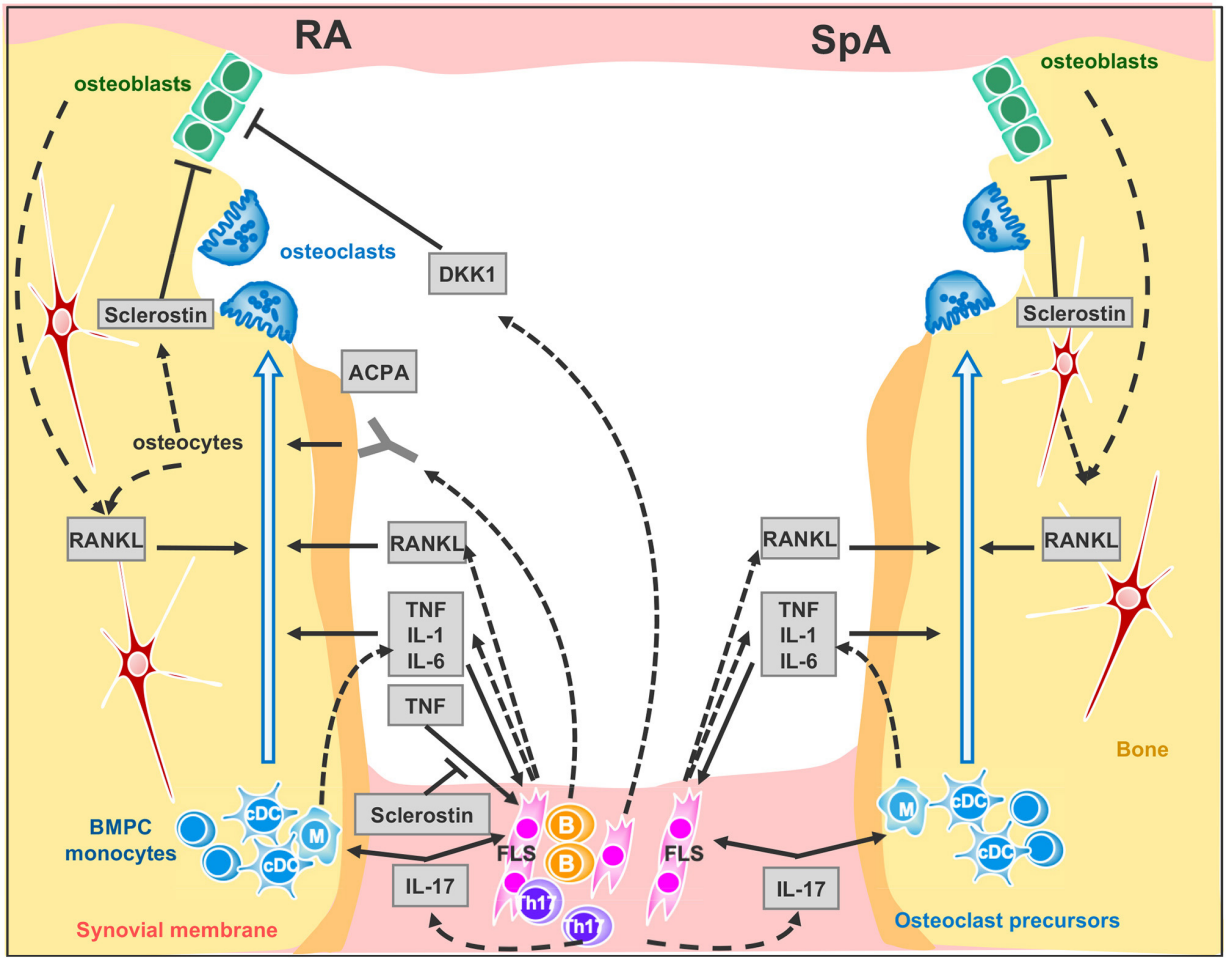
Signaling network between synovial membrane and bone in inflammatory rheumatic disease. Left panel RA and right panel SpA cytokine signaling at inflamed joint. Plain arrows indicate an action of the cytokine, factor or auto-antibodies on the cells. Dotted arrows indicate cytokine, factor or auto-antibody production by the cells. ACPA, anti-citrullinated peptide antibodies; BMPC, bone marrow progenitor cells; DKK-1, Dickkopf-1; FLS, Fibroblast-like synoviocytes. B, B cells; Th17, Th17-cells; cDC, circulating dendritic cells. M, Macrophages.

T cells have emerged as primary players through both direct and indirect mechanisms in the pathogenesis of bone loss in arthritis (39). Although osteoblasts, osteocytes and T cells express RANKL, the major RANKL-expressing cell subset in arthritic joints has been shown to be synovial fibroblasts [(39), (Figure 1)]. However, these cells express RANKL under the effect of interleukin-17 (IL-17) produced by T helper (Th) 17 cells (40). Congruent with this result, IL-17A promotes osteoclast precursor increase, bone resorption biomarker induction, and bone erosion (41, 42); its inhibition leads to improvement of inflammatory arthritis animal models (24, 43). Nevertheless, while IL-17A inhibition has demonstrated robust efficacy in SpA including PsA (44–46), it has shown only limited effect in the treatment of active RA (47–51).

IL-17-producing Th17 cells are the exclusive pro-osteoclastogenic Tcell subset while Th1 and Th2 subsets inhibit osteoclastogenesis through their respective canonical cytokines IFN-γ and IL-4 (52). Similarly, regulatory T cells inhibit osteoclastogenesis through anti-inflammatory cytokines such as IL-10 and through cytotoxic T lymphocyte antigen 4 (CTLA4) signaling, a negative regulator of T cell activation (47, 49, 50). The anti-erosive effect of abatacept, a CTLA4-Ig fusion protein efficacious in patients with RA and active PsA, underlines this effect. Deficiencies in regulatory T cell function and Th17/regulatory T cell imbalance have been identified in RA and psoriasis (53, 54). However, data on the presence and distribution of regulatory T cells in inflamed synovial tissue and on the effects of abatacept on regulatory T cell function are both limited and conflicting (8, 55–57).

## ACPA-Mediated Bone Erosion

ACPA targets are citrullinated proteins—mainly fibrinogen, α-enolase, and vimentin. Citrullination, a posttranslational conversion of arginine residues to citrulline performed by peptidylarginine deiminases, is a physiological process which can be pathologically triggered by smoking, a well-known risk factor for RA (58).

ACPA currently constitute the most specific serological marker for the diagnosis of RA and have been thereby included in the American College of Rheumatology (ACR)/European League Against Rheumatism (EULAR) 2010 RA classification criteria (59). ACPA are also a strong predictive factor for the development of bone erosion (60, 61) and can emerge long before the onset of synovitis during an initial pre-clinical phase of autoimmunity, which is either asymptomatic or only associated with arthralgia (62–65). Remarkably, the hypothesis that bone damage in RA precedes the clinical onset of disease is supported by the discovery of systemic bone loss and cortical bone erosion in a cohort of healthy ACPA-positive individuals (5), suggesting that ACPA directly trigger bone loss.

ACPA mainly belong to the IgG subtype and thus are recognized by FcγR on immune cells. It was therefore originally proposed that ACPA indirectly mediate bone loss through the enhanced production of TNF by monocytes / macrophages (66), but in recent years two groups have shown that ACPA also bind directly to citrullinated proteins on the surface of osteoclast precursors and directly enhance osteoclastogenesis (67, 68) (Figure 1). Remarkably, ACPA glycosylation patterns shift the change toward a more pro-inflammatory phenotype only within the 3 months prior to the onset of RA (69, 70). Furthermore, in newly differentiating antibody-producing cells, β-galactoside α2,6-sialyltransferase expression is regulated by Th17 cells in an IL-22- and IL-21- dependent manner, determining the glycosylation profile of IgG produced by plasma cells (70). Consequently, while IL-17 inhibition has a limited effect in the treatment of active RA, it may have a role when instituted at the early stages. Moreover, insofar as ACPA can promote bone resorption and some biologic DMARDs such as abatacept and rituximab (a monoclonal antibody against B cell CD20) can decrease ACPA levels in RA patients, the goal of achieving immunological remission with these treatments is enticing (71). However, the real value of reducing ACPA in RA patients still needs to be determined.

Taken together, these studies support a pathogenic role for ACPA in mediating bone loss in RA. In contrast, PsA is not frequently associated with circulating autoantibodies, including ACPA (72). This is probably the reason why rituximab, is effective in RA and not in PsA. However, when ACPA are present in PsA, titers are usually low but the disease phenotype is more severe with polyarticular involvement and erosive disease (73).

## Proinflammatory Cytokine-Mediated Bone Resorption

Bone loss correlates well with disease activity and severity, supporting the current therapeutic strategy in inflammatory rheumatic diseases of targeting the best control of synovitis and the biological inflammatory syndrome. Indeed, conventional DMARDs, such as methotrexate, enable protection from bone erosion simply by their ability to reduce synovitis (74). However, some RA patients in sustained clinical remission or low disease activity still continue to accrue bone erosions (38, 75), likely because of subclinical synovial inflammation (76). This evolution is probably similar in SpA, but it has not yet been clearly demonstrated in the absence of well-defined remission criteria.

TNF overexpression is sufficient to induce arthritis in mice (77). TNF operates by several mechanisms: it promotes bone resorption indirectly in conjunction with IL-6 by up-regulating RANKL expression in synovial fibroblasts (78, 79) and directly by aiding the differentiation of osteoclasts from mononuclear precursors in synovial tissues in synergy with RANKL (80) (Figure 1). Recent evidence suggests that combinations of cytokines, such as TNF plus IL-6, may drive RANK/RANKL-independent osteoclast formation (81) but this process still needs confirmation using other models. TNF also expands the pool of osteoclast precursor cells (82). Additionally, IL-1 is a mediator of TNF-induced osteoclastogenesis (83) while IL-6 is an important factor for Th17 differentiation. Accordingly, clinical trials—only in RA—with TNF blockers (16) and the Il-6 receptor blockade (84), have confirmed the impact of pro-inflammatory cytokines on osteoclastogenesis as they can retard or arrest the occurrence of bone erosion. As for the IL-1 blockade, despite having a limited effect on swelling, it protects from bone erosion in RA (85).

## Osteoformation And Erosion Repair

In RA only, the inflammatory milieu also impairs bone formation and erosion repair. TNF is the instrumental cytokine that unbalances bone homeostasis, blocking osteoblast differentiation and maturation through Wingless (Wnt) ligand signaling (86). Bone formation is governed by Wnt pathways which are critical for the osteoblast transcriptional differentiation program through the canonical β catenin-dependent activation. The Wnt ligands interact with the membrane-bound co-receptor frizzled and the low–density lipoprotein receptor-related proteins LRP-5 or LRP-6. This activated receptor complex stabilizes β catenin transcription factor, allowing its translocation to the nucleus to directly coactivate Runx2 and OPG (87, 88). In inflammatory rheumatic diseases, bone erosion repair is scarcely observed, even under biologic therapies such as TNF or IL-6 receptor blockers, and manifests only as apposition of new bone (sclerosis) at the base of the erosion (89, 90). Paradoxically, analysis of histological sections of arthritic samples, either from humans or from murine models, has shown the presence of osteoblast lineage cells close to the eroded bone once inflammation resolves (21, 91). In addition, intermittent parathyroid hormone (PTH) treatment -an anabolic agent for bone- used for treatment of osteoporosis, fails to reduce erosion volume in patients with established RA with disease activity controlled by TNF blockers (92). By contrast to humans, treatment of hTNFtg mice with a combined therapy consisting of anti-TNF together with intermittent PTH led to regression of local bone erosion and bone repair, demonstrating new bone formation (93). An alternative to anabolic treatment aiming at increasing bone formation and repair, is to block bone formation antagonists. Indeed, Wnt pro-osteogenic function is controlled and tempered by several physiological antagonists: Dickkopf proteins (DKK-1 and 2), soluble frizzled-related proteins (sFRPs) (94, 95) and sclerostin that—in the presence of Wnt ligands—antagonizes LRP-6 internalization (96, 97). In RA, TNF lessens osteoformation by up-regulating DKK-1 expression, for instance DKK-1 level is found to be elevated in RA patients' sera and in hTNFtg mice, CIA, and GPI-induced arthritis mice, (98, 99). In hTNFtg mice only, DKK-1 inhibition is able to prevent bone erosion and to promote bone formation, generating osteophytes around inflamed joints (99). Soluble frizzled-related proteins sFRP1 and sFRP2 are Wnt antagonist that sequestrate Wnt ligands, preventing them to activate frizzle/Lpr5 receptors, were also found elevated in synovial fluids of KBxN serum transfert inflammation induced mice model (91). Among the Wnt ligand antagonists, sclerostin is an attractive therapeutic target for bone loss pathologies. Sclerostin-neutralizing antibodies have been shown to have strong bone-building effects in mice, rats, monkeys, and humans (97–101). This treatment prevents the decrease of bone mineral density and bone volume at axial and appendicular sites in Collagen-Induced Arthritis mice but does not protect from erosion on the periarticular bone and fails to repair focal erosions (102). On the other hand, in hTNFtg mice, TNF induced sclerostin expression in inflammatory synoviocytes, unexpectedly, the absence of sclerostin in hTNFtg/ Sost^−/−^ mice, instead of reversing the inflammatory bone destruction, elicited exacerbation of the disease. These observations suggest that sclerostin may be involved in regulating other pathways besides Wnt signaling or has an anti-osteoclastogenic effect in TNF-dependent chronic arthritis (103). In line with this paradigm of uncovered sclerostin functions, recent findings surprisingly show that overexpressing sclerostin in murine skeletal stem cells forms overgrown bones when engrafted. This observation indicates that sclerostin could have an osteoforming effect on skeletal stem cells (104). Moreover, a recent study using non-inflammatory bone loss mouse models, unveiled a compensatory mechanism leading to increased expression of sclerostin when DKK-1 is inhibited. It would therefore perhaps be prudent before embarking upon anti-sclerostin treatments for RA, to conduct further studies in animal models of RA using *Sost* tissue-specific ablation to help obtain a better understanding of the precise role of sclerostin in chronic inflammatory diseases.

In contrast to RA, bone formation is observed in SpA at entheseal sites, resulting in endochondral bone formations. IL32γ, among others pro-inflammatory cytokines, is found elevated in SpA synovial fluid, it is proposed that IL32γ enhances osteoblast differentiation via DKK-1 suppression, thereafter promoting abnormal bone formation (105). Indeed, lower levels of DKK-1 in AS and PsA patients and sclerostin in AS patients have been reported, potentially explaining the non-impediment of osteoblast activity (99, 106, 107). In conflict with the above report, a recent meta-analysis showed no significant difference in sclerostin serum levels in AS and RA patients vs. healthy controls which suggests that sclerostin may not be associated with the pathogenesis of AS and RA (108). Last, a recent and challenging study revealed that vesicular RANK produced by mature osteoclasts stimulate early osteoblast differentiation through osteoblastic RANKL reverse signaling (109). Consequently, the development of a biological compound to trigger RANKL reverse signaling in osteoblast would be a new promising lead to promote bone formation.

## Conclusions

In inflammatory rheumatic diseases, systemic and local bone loss constitute a common key outcome in terms of functional capacity and reflects the tight interaction between the immune system and bone, leading to an increase in osteoclast activity and a consequent uncoupling of bone resorption from formation. Once established, bone erosions are at present, still irreversible. It is to be hoped that a better future understanding of the molecular pathways involved in bone loss and bone formation—particularly in the context of inflammation—will enable the development of new therapies that can selectively and directly halt, or even repair, bone erosion.

## Author Contributions

All authors listed have made a substantial, direct and intellectual contribution to the work, and approved it for publication.

### Conflict of Interest Statement

The authors declare that the research was conducted in the absence of any commercial or financial relationships that could be construed as a potential conflict of interest.
